# Supplementary material for: Efficiently accelerated bioimage analysis with NanoPyx, a Liquid Engine-powered Python framework
Source: Nat Methods. 2025 Jan 2;22(2):283–6. doi: 10.1038/s41592-024-02562-6 (PMC11810771; doi:10.1038/s41592-024-02562-6)
Supplement: Supplementary file 1 — Supplementary Tables 1–8, Notes 1–8, Fig. 1 and Methods. [file 41592_2024_2562_MOESM1_ESM.pdf]

nature methods

Supplementary information https://doi.org/10.1038/s41592-024-02562-6

Efficiently accelerated bioimage analysis with NanoPyx, a Liquid Engine-powered Python framework

In the format provided by the authors and unedited

Supplementary Table 1 – Testing Hardware Specifications

Hardware Setup	OS	CPU	RAM	GPU
1	Windows 10	Intel i9-13900k	128Gb	RTX 4090 32Gb
2	Windows 10	AMD Epyc 7F72	128Gb	NVIDIA TITAN
3	Windows 10	Intel Core i7-9700K	64Gb	NVIDIA GeForce RTX 2080 Ti
4	Ubuntu	AMD Ryzen Threadripper 2990wx	96Gb	NVIDIA GeForce GTX1070
5	Windows 10	AMD Ryzen 7 3700X 8 core	32Gb	NVIDIA GeForce GTX 1660 Ti
6	MacOS	M2 Pro	16Gb	Integrated
7	MacOS	M1 Pro	16Gb	Integrated
8	MacOS	M1	16Gb	Integrated
9	Windows 10	Intel i5-7300HQ	8Gb	NVIDIA GeForce GTX1060 Mobile
10	Ubuntu	AMD Ryzen 7 4700U	12Gb	Integrated

Supplementary Table 2 – 2D Convolution benchmarking across multiple hardware using 3 different conditions

Task: 2D Convolution

Slow → Fast   Hardware Limitation

Hardware Setup	Image size: 500 x 500 Kernel size: 5 x 5			Image size: 2500 x 2500 Kernel size: 9 x 9			Image size: 2500 x 2500 Kernel size: 21 x 21		
	OpenCL	Threaded	Unthreaded	OpenCL	Threaded	Unthreaded	OpenCL	Threaded	Unthreaded
1	0.112	0.002	0.011	0.101	0.042	0.527	0.102	0.186	2.267
2	0.244	0.004	0.022	0.158	0.063	0.994	0.199	0.258	4.433
3	0.323	0.003	0.013	0.134	0.153	0.832	0.157	0.640	3.876
4	0.171	0.004	0.007	0.186	0.040	0.562	0.233	0.135	2.690
5	0.227	0.004	0.020	0.144	0.162	0.865	0.191	0.702	3.831
6	0.025	0.002	0.004	0.055	0.069	0.431	0.096	0.431	3.080
7	0.036	0.002	0.005	0.046	0.086	0.458	0.097	0.528	3.271
8	0.034	0.002	0.005	0.058	0.094	0.465	0.131	0.594	3.351
9	0.244	0.020	0.053	0.247	1.180	2.222	0.300	5.720	9.699
10		0.010	0.009		0.164	0.577		0.574	2.909

Supplementary Table 3 – Catmull-rom interpolation benchmarking across multiple hardware using 3 different conditions

Task: Catmull-Rom Interpolation

Slow → Fast   Hardware Limitation

Hardware Setup	Image size: 10 x 10 x 10 Magnification: 5			Image size: 10 x 100 x 100 Magnification: 5			Image size: 10 x 300 x 300 Magnification: 5		
	OpenCL	Threaded	Unthreaded	OpenCL	Threaded	Unthreaded	OpenCL	Threaded	Unthreaded
1	0.119	0.000	0.001	0.083	0.008	0.119	0.272	1.001	8.794
2	0.196	0.001	0.002	0.126	0.013	0.190	0.516	0.856	14.60
3	0.154	0.000	0.001	0.107	0.020	0.145	0.590	1.822	12.77
4	0.185	0.012	0.002	0.147	0.029	0.229	0.982	12.44	20.77
5	0.173	0.001	0.002	0.128	0.027	0.174	0.661	1.627	12.92
6	0.020	0.001	0.001	0.010	0.016	0.063	0.465	1.523	5.678
7	0.013	0.001	0.001	0.013	0.021	0.067	0.809	1.885	6.083
8	0.014	0.001	0.001	0.014	0.020	0.068	1.545	1.903	6.132
9	0.208	0.007	0.002	0.127	0.108	0.280	1.008	9.909	22.96
10		0.009	0.002		0.067	0.217		3.710	19.33

Supplementary Table 4 – Non-local means denoising benchmarking across multiple hardware using 3 different conditions

Task: Non-Local Means Denoising

Slow → Fast; Hardware Limitation

Hardware Setup	Image size: 1 x 200 x 200 patch size: 5, patch distance: 10			Image size: 1 x 500 x 500 patch size: 5, patch distance: 100			Image size: 1 x 1000 x 1000 patch size: 50, patch distance: 50		
	OpenCL	Threaded	Unthreaded	OpenCL	Threaded	Unthreaded	OpenCL	Threaded	Unthreaded
1	0.109	0.028	0.068	0.560	12.95	40.99	50.83	691.9	28.77
2	0.129	0.038	0.117	1.402	15.69	73.77	150.6	645.9	52.45
3	0.120	0.098	0.085	1.467	46.25	68.61	245.0	3066	52.86
4	0.181	0.043	0.125	3.358	10.29	99.20	386.0	863.7	70.94
5	0.134	0.098	0.111	3.806	39.85	65.68	443.8	1648	47.06
6	0.022	0.061	0.107	5.471	28.17	88.33		781.2	63.76
7	0.026	0.070	0.112	8.531	32.31	88.94		943.5	64.69
8	0.043	0.184	0.118	11.68	54.14	90.68		1585	65.54
9	0.149	0.396	0.178	5.767	191.7	113.5	1094	11234	82.41
10		0.098	0.131		55.86	103.4		4350	73.65

Supplementary Table 5 – Jupyter Notebooks available as part of NanoPyx

#	Notebook	Type	Link
1	Liquid Engine Runtimes improvement	Showcase	https://github.com/HenriquesLab/NanoPyx/blob/main/src/scripts/publication/LiquidImprovement.ipynb
2	Liquid Engine Benchmarking	Showcase	https://github.com/HenriquesLab/NanoPyx/blob/main/notebooks/LiquidEngineBenchmarking.ipynb
3	Liquid Engine's Fuzzy logic	Showcase	https://github.com/HenriquesLab/NanoPyx/blob/main/src/scripts/publication/FuzzyLogic.ipynb
4	Liquid Engine Overhead	Showcase	https://github.com/HenriquesLab/NanoPyx/blob/main/src/scripts/publication/Overhead.ipynb
5	NanoPyx methods using simple Python calls	Showcase	https://github.com/HenriquesLab/NanoPyx/blob/main/src/scripts/publication/TestingMethods.ipynb
6	Example Notebook	"Codeless"	https://github.com/HenriquesLab/NanoPyx/blob/main/notebooks/ExampleDataSRRFandQC.ipynb
7	Drift Correction	"Codeless"	https://github.com/HenriquesLab/NanoPyx/blob/main/notebooks/DriftCorrection.ipynb
8	Channel Registration	"Codeless"	https://github.com/HenriquesLab/NanoPyx/blob/main/notebooks/ChannelRegistration.ipynb
9	Image fidelity and resolution metrics	"Codeless"	https://github.com/HenriquesLab/NanoPyx/blob/main/notebooks/SRMetrics.ipynb
10	Non-local Means Denoising	"Codeless"	https://github.com/HenriquesLab/NanoPyx/blob/main/notebooks/NonLocalMeansDenoising.ipynb
11	SRRF	"Codeless"	https://github.com/HenriquesLab/NanoPyx/blob/main/notebooks/SRRFandQC.ipynb
12	eSRRF	"Codeless"	https://github.com/HenriquesLab/NanoPyx/blob/main/notebooks/eSRRFandQC.ipynb

Supplementary Table 6 – Comparison of NanoPyx and NanoJ available methods, and which NanoPyx methods are using the Liquid Engine.

	Available in:		
**Method Name**	**NanoPyx**	**Through Liquid Engine**	**NanoJ**
Drift Correction	Yes	Yes	Yes
Channel Registration	Yes	Yes	Yes
SRRF	Yes	Yes	Yes
eSRRF	Yes	Yes	Yes
NanoJ-SQUIRREL error map	Yes	In development	Yes
Fourier Ring Correlation	Yes	In development	Yes
Image Decorrelation Analysis	Yes	In development	No
Interpolations	Yes	Yes	No
Image Shift	Yes	Yes	No
Image Resize	Yes	Yes	No
Image Rescale	Yes	Yes	No
Image Rotation	Yes	Yes	No
2D Convolution	Yes	Yes	No
Non-local Means Denoising	Yes	Yes	No

Supplementary Table 7 – Supporting Video Tutorials

#	Title	Link
1	What is NanoPyx?	https://youtu.be/iAdgusBAU0Q
2	How to use NanoPyx in Google Colab	https://youtu.be/KD0RzolFnd4
3	How to install NanoPyx and run Jupyter Notebooks locally	https://youtu.be/Dx2lHoRB044
4	How to build your Liquid Engine class in 1 minute	https://youtu.be/QQsXrZ_jFa8

5	How to Benchmark your Implementations with the Liquid Engine in 1 minute	https://youtu.be/9hF7nLtFzoo
6	How to create a Python package with the Liquid Engine	https://youtu.be/s2SY6IlsWQI

Supplementary Table 8 – Example datasets used in this publication.

Label	Description	Data Shape	Link
a549_mt	Fixed A549 cells immunolabelled against ß-tubulin with Alexa Fluor™ 647, acquired with the Oxford Nanoimager (ONI)	10000x283x283	Available in Zenodo: https://zenodo.org/record/8318395
huvec_nuclei	HUVEC stained with DAPI, acquired in a Marianas spinning-disk confocal microscope.	100x512x512	

Supplementary Note 1: How computational acceleration for an algorithm implementation written with Cython, PyOpenCL and Numba is achieved.

Bioimage analysis frameworks are an important part of life sciences research and have significantly empowered researchers in their work. With the adoption of deep learning techniques, a lot of bioimage analysis workflows have been developed using Python. Even though Python is an interpreted, high-level programming language that allows rapid development and easy code readability, its flexibility and dynamic nature come at the cost of performance speed. Operations in pure Python are generally slower compared to compiled languages like C/C++. There are several methods to accelerate Python code by bypassing the Global Interpreter Lock (GIL) and compilation to machine code. Three popular approaches are Cython[1], PyOpenCL[2] and Numba[3]:

- Cython[1] is a static compiler that converts Python code into optimised C/C++ code that can be compiled into a Python extension module. It provides Python-like syntax while supporting calling C functions and declaring C types. Cython[1] code runs significantly faster than Python because it bypasses the GIL to allow multi-threading and performs low-level optimisations like loop unrolling. One limitation is that Cython[1] requires explicit type declarations, which removes some of Python's dynamism.
- PyOpenCL[2] allows Python code to execute parallel computations on Graphical Processing Units (GPUs) through the OpenCL[4] framework. Computational tasks are offloaded to the GPU, which has thousands of tiny processing cores suited for data-parallel operations. PyOpenCL[2] translates Python functions into OpenCL[4] kernels that run efficiently on GPUs. This offers massive parallelism and speedup compared to Python limited by single-CPU execution. Despite PyOpenCL[2] not requiring a physical GPU to run, the best and easiest performance improvement requires one, which can be a limiting factor for some users.
- Numba[3] is a just-in-time (JIT) compiler that converts Python functions into optimised machine code using the LLVM compiler framework. It is designed to accelerate numerical and scientific workloads using NumPy[5] arrays and math operations. Numba[3]-compiled code avoids interpreter overhead and leverages vectorisation, loop-unrolling and parallel execution on multicore CPUs. But Numba[3] has compilation overhead on the first run.

All these three tools can significantly accelerate Python code by bypassing interpreter overhead and utilizing efficient compilation, parallelism, and hardware optimisation. Cython[1] translates Python to C/C++ code that can multi-thread and leverage CPU efficiency. PyOpenCL[2] taps into massively parallel GPU hardware. Numba[3] optimises machine code for numerical workloads on CPUs. Typical speedups depend on the methods used, nature of the code, size of the input data and hardware used.

Depending on the hardware, some of these approaches might not provide the expected acceleration. These hardware barriers can happen, for example, when the memory available on the hardware is not enough for the requested computation, and

the algorithm simply fails to run. Another example is running OpenCL kernels through a CPU's integrated graphics instead of a native GPU. Although this could lead to a speed improvement over other approaches, throughout our testing we found that in some hardware combinations, some computationally and memory intensive algorithms can silently fail yielding wrong results.

Aiming to be agnostic of user hardware, NanoPyx tries to deal with these barriers by providing several approaches for acceleration. It is the responsibility of the Liquid Engine and its agent to choose the fastest. Upon benchmarking an algorithm, some implementations might be disabled by the agent if their output is different than all other implementations, signifying a hardware barrier. This is done on a case-by-case basis, specific for the set of parameters for which it failed, allowing the implementation to be used elsewhere and/or with a different set of parameters.

Supplementary Note 2: Parallelization within different implementations – NLM denoising example

Among all implementations a recurrent theme is parallelization. How parallelization is achieved is intrinsically algorithm dependent. For example, in NanoPyx, upscaling and downscaling operations and their associated interpolators, are parallelized in a pixel-by-pixel manner. This is possible since the calculation of each pixel value in the final image is independent of all other pixels.

Some image analysis methods lend themselves very well to a parallel architecture whilst others don't. We would like to highlight non-local means denoising as an example method that showcases very different behaviours depending on how the algorithm is parallelized. Our non-local means denoising implementations within the Liquid Engine are based upon the well-established scikit-image Python package from which we decided to keep the name of the user-defined parameters.

Non-local means denoising, is a denoising algorithm that replaces each pixel by an average of all other pixels that have a similar neighbourhood. The method requires at least two parameters that are user-defined. The patch size defines the size of the neighbourhood of each pixel. The patch distance defines the maximum distance relative to the centre of each patch the algorithm searches for similar patches.

There are two distinct ways to write an algorithm that calculates non-local means denoising which we decided to call "pixel" and "patch".

The "pixel" non-local means denoising is the more intuitive of the two and is akin to the "fast_mode=False" implemented in scikit-image and originally based on the work of Buades *et al*[6]. In this implementation we transverse each pixel of the input image and for each one of them check all the pixels closer than patch distance for other pixels with a similar neighbourhood. This method is easily parallelizable using conventional approaches since there is a clear independence between the results of each pixel. However, its computational complexity increases very quickly with increasing patch size, patch distance and image size.

The ”patch” non-local means denoising is akin to the “fast_mode=True” in scikit-image and originally based on the work of Darbon *et al.*[7]. This implementation denoises the image by reparametrizing the original formulation to work with integrals of the squared difference of the image and its shift by a fixed value. The integral images are then used to calculate how each pixel in the original image must change. This yields an algorithm where its computational complexity is much less sensible to patch size. Although much more time-efficient this approach uses twice as much memory as the previous one. Although possible to parallelize, the increase in the memory footprint coupled to the fact that the result of each pixel depends on the integral images of several shifts makes this approach much less amenable to parallelization.

Within NanoPyx, both the “patch” and “pixel” approaches have threaded and unthreaded implementations. However, most of these are hidden to the end-user. Throughout our testing in different hardware setups, we found that the threaded implementations of the “patch” non-local means denoising were considerably slower than unthreaded versions. The additional memory and memory bandwidth that was required slowed down the method considerably. Even so, the reduction in computational complexity is great enough that even if not threaded, it can be faster than threaded “pixel” approaches for some cases (Supplementary Figure 1).

As such, for the end-users, NanoPyx exposes only a fraction of these implementations. For ease of use, only one public non-local means denoising method is available, but it uses the “patch” approach for its unthreaded implementation whilst all threaded and GPU based implementations use the “pixel” approach.

Supplementary Note 3: Naming each implementation.

NanoPyx aims to provide several implementations for each algorithm. Most commonly the different implementations differ on what physical device the algorithm is being run and how the device is being exploited.

Particularly, we define the following implementations:

1. CPU Unthreaded: Cython based implementation that is compiled and that runs on a single CPU core.
2. CPU Threaded: Cython based implementation that is compiled and that runs on multiple CPU cores. Multiple threaded implementations can coexist by changing the scheduler behind the thread organization (Static, Guided and Dynamic).
3. GPU (OpenCL): OpenCL implementation that is passed through the PyOpenCL wrapper library and runs on a GPU.
4. Python: Python based implementation that is usually not compiled and that runs on a single CPU core.
5. Numba[3], Transonic[8], Dask[9], etc: these are all the other implementations with self-explanatory names that rely on external libraries for their optimization.

These can potentially be run on either CPU or GPU, with a single or multiple cores.

Supplementary Note 4: Metaprogramming in the Liquid Engine

Metaprogramming is a programming technique where a program can manipulate or generate code during runtime. In NanoPyx, metaprogramming is used to generate implementations of the same task semi-automatically.
Metaprogramming in the Liquid Engine is implemented with Mako[10] templates.
Mako is a Python-based template library. It allows the user to create template files that can be parametrized by the user and then rendered at runtime. In short, a user can build a template where its output is dependent on user defined parameters or where its output is modified by Python code written inside the template file. The Mako library also allows for template inheritance, chaining, and other more complex features.
Within the Liquid Engine the use of Mako templates allows the developers to avoid manual coding by propagating repetitive code into multiple specialised implementations of the same algorithm. A specific example within the Liquid Engine is writing only the single-threaded version of an algorithm (e.g. an interpolation) and using it as a template to implement several multi-threaded versions with different schedulers. A specific example is found in the following code snippet:

```
<%!
schedulers = ['unthreaded','threaded','threaded_guided','threaded_dynamic','threaded_static']
%>

% for sch in schedulers:
def _run_${sch}():
    % if sch=='unthreaded':
    for r in range(10):
    % elif sch=='threaded':
    for r in prange(10):
    % else:
    for r in prange(10,schedule="${sch.split('_')[1]}"):
    % endif
        pass

% endfor
```

If rendered, a template with this code would show 5 different realizations of the same function, with different names and different for-loop parallelization.

```python
def _run_unthreaded():
    for r in range(10):
        pass

def _run_threaded():
    for r in prange(10):
        pass

def _run_threaded_guided():
    for r in prange(10,schedule="guided"):
        pass

def _run_threaded_dynamic():
    for r in prange(10,schedule="dynamic"):
        pass

def _run_threaded_static():
    for r in prange(10,schedule="static"):
        pass
```

Using Mako templating we can also enclose helpful scripting tools that can be used while rendering the template. We do this with the c2cl tool. c2cl is specifically designed to extract and process C functions and propagate them into the necessary .cl files so they can be used in OpenCL kernels. Manual conversion of these code snippets can be time-consuming and prone to errors. With this tool we automate the process of porting C code to OpenCL by extracting reusable code blocks from the C functions and inserting the modified kernels back into an OpenCL file.
Using a Mako template inside NanoPyx entails writing a template file in the *mako_templates* folder. The name of the file should represent the relative path where the rendered template should live. All templates are rendered at compile time.
We want to highlight the use of metaprogramming to maintain consistency across various code implementations. Modifications to the code can be ensured to be seamlessly and consistently applied to all relevant implementations. Developers can effectively manage code updates and improvements whilst reducing redundancy and enhancing code maintainability.
Whilst the Mako template library was technically written with web development in mind, it is not a novel concept to use template libraries of this sort to develop code with scientific purposes in mind. A prominent example is the PyOpenCL[2] library, which NanoPyx also uses, where Mako can be used to generate OpenCL kernels that can be parametrized and dynamically changed at runtime.

Supplementary Note 5: The machine learning basis of the Liquid Engine

The NanoPyx Liquid Engine presents a straightforward machine learning technique for performance self-tuning. To do so, it logs execution times of operations across multiple implementations like Python, Numba[3], Cython[1] and OpenCL[4]. These

benchmarking times are used to train basic regression models to predict the occurrence of delays. If delays are detected the trained models are used at runtime to estimate the magnitude and occurrence of a delay in a specific implementation. Over time, the benchmarking data is aggregated to refine the models continuously. This allows the Liquid Engine to "learn" the optimal implementations for a given platform, device, and data shape to maximise performance. It's important to note that these principles do not use neural networks, rather focusing on looped data-driven optimisations. In summary, fundamental machine learning principles are applied in the engine itself for auto-tuning - the methods exposed to users are traditional image processing functions using optimised implementations under the hood.

Supplementary Note 6: Liquid Engine's Fuzzy logic

The Liquid Engine employs fuzzy logic[11] to match a specific function call to its most similar past benchmark.

The Liquid Engine adaptive nature is highly dependent on the existence of appropriate benchmarks for each implementation. As previously demonstrated, the time it takes for a particular image analysis task to execute is greatly influenced by the size of the input image and by the parameters chosen to perform the given task. To address this variability, benchmarks are stored separately for each unique set of parameters and data size. This approach allows the Liquid Engine to dynamically adjust its performance based on the specific conditions of each task, resulting in more accurate and optimised outcomes.

When the Agent receives a request to execute a method using the Liquid Engine, it checks its records for historical runtime data. However, when dealing with a new method executed with a unique combination of parameters, no existing benchmarks are available. To address this, the Agent employs the following strategy: it searches for the most similar set of parameters that has been previously used. This is because each set of parameters is associated with a runtime score, which was saved along with the historical runtime data in benchmark files. This score is determined by considering various factors, such as the dimensions of the input image and other relevant parameters. By finding the most similar parameters with known benchmark data, the Agent leverages this score to estimate and adapt the expected runtime performance for the new method, even in the absence of specific benchmarks. This adaptive approach allows the Liquid Engine to intelligently adjust its behaviour and make informed decisions when executing methods with varying input conditions.

Finally, if no appropriate benchmarks exist for a specific run type, the score of the current parameters is compared to the score of all other benchmarks, and the benchmarks with the closest score are used.

An example of the fuzzy logic in action can be seen on the Fuzzy Logic Jupyter Notebook (Supplementary Table 2, “Liquid Engine's Fuzzy Logic).

Supplementary Note 7: Example datasets in NanoPyx

NanoPyx provides users a wide range of example datasets. These datasets not only draw from our previous publications but also tap into publicly available datasets[12]. These were integrated into the NanoPyx framework to facilitate testing and development, offering users an opportunity to gain hands-on experience and explore the capabilities of the library.

These include single-molecule localization microscopy data of Cos7 cells expressing Utrophin-GFP[13]; U2OS with microtubules labelled with AF647; Jurkat T cells expressing LifeAct-GFP[14]; Structured Illumination Microscopy data of VACV A4 virions[15]; among others.

The management and loading of datasets within NanoPyx are orchestrated through a class specifically designed for data management, facilitating efficient access and use. The class provides functions to list datasets and retrieve their information, enabling users to effortlessly identify and choose relevant datasets.

Most of the datasets are stored and accessed via Google Drive and can be automatically downloaded as zip files.

Once downloaded, these zip files can be effortlessly converted into numpy arrays, a process that seamlessly manages the complexities of image retrieval and manipulation.

In Jupyter or Colab notebooks, users can easily access the datasets through a user-friendly graphical interface (GUI) that has been developed to streamline the process. This interface empowers users to select datasets from a diverse array of options, all of which are named to provide clear context. This intuitive approach ensures efficient dataset selection and integration into analysis workflows.

Supplementary Note 8: Implementing your own Liquid Engine methods

We have created a project template using cookiecutter[16]. Cookiecutter is a Python package developed as a command-line utility that helps generate Python package projects. The Liquid Engine cookiecutter is a remote template hosted on github that any user can take advantage of to generate a Python package which implements a custom-made Liquid Engine method, with NanoPyx as a dependency. The use of cookiecutter allows each user to customise the generated package during its creation. The generated Python package is fully functional, but barebones in order to allow the user to quickly change it as they see fit. The cookiecutter template can be found here: https://github.com/HenriquesLab/LiquidEngineCookieCutter.

A

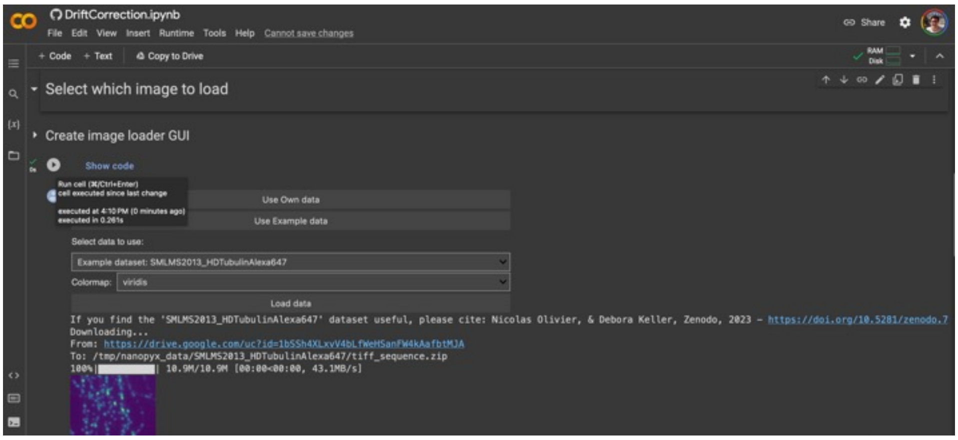

B

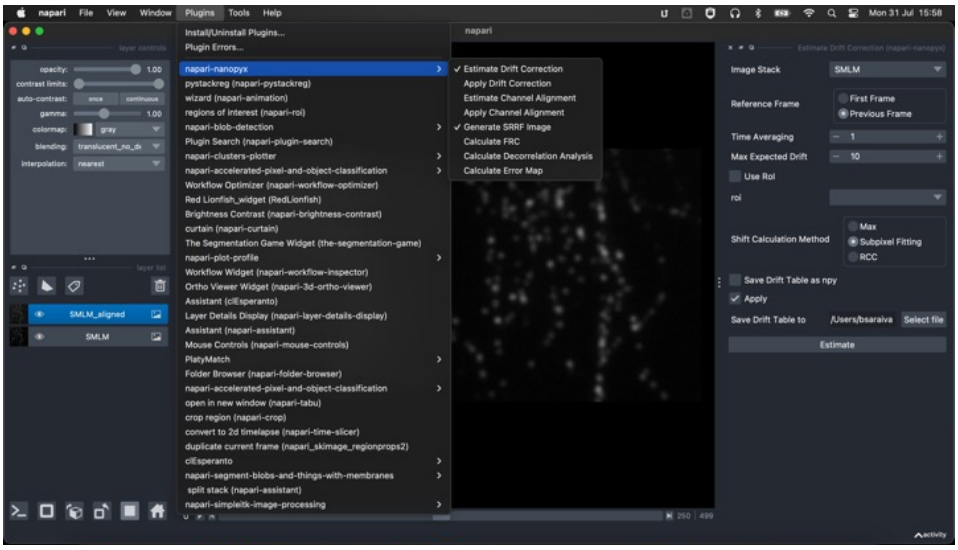

Supplementary Figure 1 – NanoPyx is available to users independently of their coding expertise. Besides using NanoPyx as a Python library, users also have access to Jupyter notebooks[17] (A) that can either be run locally or through Google Colaboratory. Additionally, users have access to a napari[18] plugin (B) with the implemented methods.

Supplementary Methods – Use of Large Language Models. The authors acknowledge the use of large language models for improving the accuracy of spelling and rephrasing sentences to enhance readability.

References

1. Behnel, S. *et al.* Cython: The Best of Both Worlds. *Comput. Sci. Eng.* **13**, 31–39 (2011).
2. Kloeckner, A. *et al.* PyOpenCL. Zenodo https://doi.org/10.5281/zenodo.7063192 (2022).
3. Lam, S. K., Pitrou, A. & Seibert, S. Numba: a LLVM-based Python JIT compiler. in *Proceedings of the Second Workshop on the LLVM Compiler Infrastructure in HPC* 1–6 (ACM, Austin Texas, 2015). doi:10.1145/2833157.2833162.
4. Stone, J. E., Gohara, D. & Shi, G. OpenCL: A Parallel Programming Standard for Heterogeneous Computing Systems. *Comput. Sci. Eng.* **12**, 66–73 (2010).
5. Harris, C. R. *et al.* Array programming with NumPy. *Nature* **585**, 357–362 (2020).
6. A non-local algorithm for image denoising | IEEE Conference Publication | IEEE Xplore. https://ieeexplore.ieee.org/document/1467423.
7. Darbon, J., Cunha, A., Chan, T. F., Osher, S. & Jensen, G. J. Fast nonlocal filtering applied to electron cryomicroscopy. in *2008 5th IEEE International Symposium on Biomedical Imaging: From Nano to Macro* 1331–1334 (2008). doi:10.1109/ISBI.2008.4541250.
8. Transonic: Make your Python code fly at transonic speeds! FluidDyn (2023).
9. Rocklin, M. Dask: Parallel Computation with Blocked algorithms and Task Scheduling. in 126–132 (Austin, Texas, 2015). doi:10.25080/Majora-7b98e3ed-013.
10. Mako : Templates for Python | BibSonomy. https://www.bibsonomy.org/bibtex/aa47d818a1c2f889b7456117003b3d42.
11. Novak, V., Perfiljeva, I. & Mockor, J. Mathematical Principles of Fuzzy Logic. in (1999). doi:10.1007/978-1-4615-5217-8.

12. Olivier, N. & Keller, D. STORM Vectashield datasets (Tubulin). Zenodo https://doi.org/10.5281/zenodo.7620025 (2023).

13. Culley, S., Tosheva, K. L., Matos Pereira, P. & Henriques, R. SRRF: Universal live-cell super-resolution microscopy. *Int. J. Biochem. Cell Biol.* **101**, 74–79 (2018).

14. Gustafsson, N. *et al.* Fast live-cell conventional fluorophore nanoscopy with ImageJ through super-resolution radial fluctuations. *Nat. Commun.* **7**, 12471 (2016).

15. Gray, R. D. M. *et al.* VirusMapper: open-source nanoscale mapping of viral architecture through super-resolution microscopy. *Sci. Rep.* **6**, 29132 (2016).

16. Greenfeld, Audrey Roy. cookiecutter.

17. Kluyver, T. *et al.* Jupyter Notebooks – a publishing format for reproducible computational workflows. in *Positioning and Power in Academic Publishing: Players, Agents and Agendas* 87–90 (IOS Press, 2016). doi:10.3233/978-1-61499-649-1-87.

18. Sofroniew, N. *et al.* napari: a multi-dimensional image viewer for Python. Zenodo https://doi.org/10.5281/zenodo.7276432 (2022).
