# Supplementary material for: Efficiently accelerated bioimage analysis with NanoPyx, a Liquid Engine-powered Python framework
Source: Nat Methods. 2025 Jan 2;22(2):283–6. doi: 10.1038/s41592-024-02562-6 (PMC11810771; doi:10.1038/s41592-024-02562-6)
Supplement: Supplementary file 2 — Reporting Summary [file 41592_2024_2562_MOESM2_ESM.pdf]

nature portfolio

Corresponding author(s): Ricardo Henriques
Last updated by author(s): Oct 10, 2024

Reporting Summary

Nature Portfolio wishes to improve the reproducibility of the work that we publish. This form provides structure for consistency and transparency in reporting. For further information on Nature Portfolio policies, see our Editorial Policies and the Editorial Policy Checklist.

Statistics

For all statistical analyses, confirm that the following items are present in the figure legend, table legend, main text, or Methods section.

n/a	Confirmed	
☒	☐	The exact sample size (*n*) for each experimental group/condition, given as a discrete number and unit of measurement
☒	☐	A statement on whether measurements were taken from distinct samples or whether the same sample was measured repeatedly
☒	☐	The statistical test(s) used AND whether they are one- or two-sided<br>*Only common tests should be described solely by name; describe more complex techniques in the Methods section.*
☒	☐	A description of all covariates tested
☒	☐	A description of any assumptions or corrections, such as tests of normality and adjustment for multiple comparisons
☒	☐	A full description of the statistical parameters including central tendency (e.g. means) or other basic estimates (e.g. regression coefficient) AND variation (e.g. standard deviation) or associated estimates of uncertainty (e.g. confidence intervals)
☒	☐	For null hypothesis testing, the test statistic (e.g. F, t, r) with confidence intervals, effect sizes, degrees of freedom and P value noted<br>*Give P values as exact values whenever suitable.*
☒	☐	For Bayesian analysis, information on the choice of priors and Markov chain Monte Carlo settings
☒	☐	For hierarchical and complex designs, identification of the appropriate level for tests and full reporting of outcomes
☒	☐	Estimates of effect sizes (e.g. Cohen's d, Pearson's r), indicating how they were calculated

Our web collection on statistics for biologists contains articles on many of the points above.

Software and code

Policy information about availability of computer code

Data collection	Images were collected as indicated in the material and methods. All the instruments used are commercially available and were controlled using the software provided by the manufacturer.
Data analysis	Images used in the manuscript were processed with NanoPyx 0.6.1, Napari 0.4.19 or Fiji 1.54f as indicated.<br>NanoPyx is can be accessed from our GitHub page https://github.com/HenriquesLab/NanoPyx. The Liquid Engine is available through its github repository page https://github.com/HenriquesLab/LiquidEngine/. These resources are fully open-source, providing users with tutorials, Jupyter Notebooks for Google Colab and many real-life example datasets for training and testing.

For manuscripts utilizing custom algorithms or software that are central to the research but not yet described in published literature, software must be made available to editors and reviewers. We strongly encourage code deposition in a community repository (e.g. GitHub). See the Nature Portfolio guidelines for submitting code & software for further information.

Data

Policy information about availability of data

All manuscripts must include a data availability statement. This statement should provide the following information, where applicable:

- Accession codes, unique identifiers, or web links for publicly available datasets
- A description of any restrictions on data availability
- For clinical datasets or third party data, please ensure that the statement adheres to our policy

The example datasets we use to showcase NanoPyx are available for download in Zenodo (links provided in Supplementary Table S1 and our GitHub page).

Human research participants

Policy information about studies involving human research participants and Sex and Gender in Research.

Reporting on sex and gender	N/A
Population characteristics	N/A
Recruitment	N/A
Ethics oversight	N/A

Note that full information on the approval of the study protocol must also be provided in the manuscript.

Field-specific reporting

Please select the one below that is the best fit for your research. If you are not sure, read the appropriate sections before making your selection.

☒ Life sciences ☐ Behavioural & social sciences ☐ Ecological, evolutionary & environmental sciences

For a reference copy of the document with all sections, see nature.com/documents/nr-reporting-summary-flat.pdf

Life sciences study design

All studies must disclose on these points even when the disclosure is negative.

Sample size	We have used 2 biological datasets, described in the data availability section, and images simulated on the fly, available in the jupyter notebooks described in the code availability section, to generate the benchmarks of different methods
Data exclusions	No data was excluded
Replication	As images were only used as examples of the type of image that can be run in the methods no replication was needed.
Randomization	As images were only used as examples of the type of image that can be run in the methods no randomization was needed.
Blinding	As images were only used as examples of the type of image that can be run in the methods no blinding was needed.

Reporting for specific materials, systems and methods

We require information from authors about some types of materials, experimental systems and methods used in many studies. Here, indicate whether each material, system or method listed is relevant to your study. If you are not sure if a list item applies to your research, read the appropriate section before selecting a response.

Materials & experimental systems

n/a	Involved in the study	
☐	☒	Antibodies
☐	☒	Eukaryotic cell lines
☒	☐	Palaeontology and archaeology
☒	☐	Animals and other organisms
☒	☐	Clinical data
☒	☐	Dual use research of concern

Methods

n/a	Involved in the study	
☒	☐	ChIP-seq
☒	☐	Flow cytometry
☒	☐	MRI-based neuroimaging

Antibodies

Antibodies used

Anti-α-Tubulin antibody, Mouse monoclonal, clone DM1A (Sigma, Catalog #T6199) (1:250); Anti-Tubulin-α Antibody, Mouse monoclonal, clone 10D8 (BioLegend, Catalog #625901) (1:500); Anti-Human Septin 7 IgG, Rabbit Polyclonal, (IBL, Catalog # JP18991) (1:100); Anti-Tubulin Beta 3 (TUBB3), Mouse monoclonal, clone AA10 (BioLegend, Catalog#657401) (1:500); Conjungated F(ab')2-Goat anti-Mouse IgG – Alexa Fluor 647 (ThermoFisher, Catalog #A-21237) (1:200); Conjugated Goat anti-Rabbit IgG - Alexa Fluor™ 555 (ThermoFisher Catalog #A-21428) (1:200).

Validation

Anti-α-Tubulin antibody (Catalog #T6199). Isotype: IgG1. Verified reactivity (VR): yeast, mouse, amphibian, human, rat, chicken, fungi, bovine. Antibody Type (AT): Monoclonal. Host species: Mouse. Concentration 1mg/ml. Application in immunoblotting, immunocytochemistry, immunofluorescence radioimmunoassay and western blot. Independent enhanced validation: antibody specificity demonstrated using multiple antibodies against target in immunohistochemistry or immunocytochemistry.
Anti-Tubulin-α Antibody (Catalog #625901). Isotype: Mouse IgM, κ. VR: Human, mouse, rat and all species. AT: monoclonal. Host species: Mouse. Concentration 0.5mg/ml. Application in western blotting (quality tested), immunohistochemistry – Paraffin and immunocytochemistry.
Anti-Human Septin 7 IgG (IBL, Catalog #JP18991). Isotype: IgG. VR: Human, mouse and rat. AT: Polyclonal. Host species: Rabbit. Concentration 0.1mg/ml. Application in western blotting, immunohistochemistry and immunoprecipitation.
Anti-Tubulin Beta 3 (TUBB3) (BioLegend, Catalog #657401). Isotype: Mouse IgG2a. VR: Mouse, Rat, Human. AT: Monoclonal. Host species: mouse. Concentration: 0.5mg/ml. Application in western blotting (quality tested), immunocytochemistry (verified), flow cytometry, immunofluorescence microscopy and spatial biology (IBEX). Knock-out validated.
Conjugated F(ab')2-goat anti-mouse IgG – Alexa Fluor 647 (ThermoFisher, Catalog #A-21237). Isotype: IgG. VR: Mouse. AT: Polyclonal. Host species: Goat/IgG. Concentration 2mg/ml. Application in western blotting, immunohistochemistry and immunocytochemistry. Cross adsorbed: against human IgG and serum.
Conjugated Full antibody-Goat anti-Rabbit IgG - Alexa Fluor 555 (ThermoFisher Catalog #A-21428); Isotype: IgG. VR: Rabbit. AT: Polyclonal. Host species: Goat/IgG. Concentration: 2mg/ml. Application in immunohistochemistry, immunocytochemistry and flow cytometry. Cross adsorbed: against human IgG, human serum, mouse IgG, mouse serum and bovine serum.

Eukaryotic cell lines

Policy information about cell lines and Sex and Gender in Research

Cell line source(s)	A549 cell line (The European Coollection of Authenticated Cell Cultures (from ECACC Catalog # 86012804); Human Umbilical Vein Endothelial Cells (HUVEC) (from PromoCell C-12203)
Authentication	Cell lines were not authenticated
Mycoplasma contamination	Cells lines used tested negative for Mycoplasma contamination
Commonly misidentified lines (See ICLAC register)	None
